# The Link between Individual Personality Traits and Criminality: A Systematic Review

**DOI:** 10.3390/ijerph18168663

**Published:** 2021-08-17

**Authors:** N. K. Tharshini, Fauziah Ibrahim, Mohammad Rahim Kamaluddin, Balan Rathakrishnan, Norruzeyati Che Mohd Nasir

**Affiliations:** 1 Faculty of Social Sciences and Humanities, Universiti Malaysia Sarawak, Kota Samarahan 94300, Sarawak, Malaysia; 2 Centre for Research in Psychology and Human Well-Being, Faculty of Social Sciences and Humanities, Universiti Kebangsaan Malaysia, Bangi 43600, Selangor, Malaysia; ifauziah@ukm.edu.my (F.I.); rahimk@ukm.edu.my (M.R.K.); 3 Faculty of Psychology and Education, Universiti Malaysia Sabah, Kota Kinabalu 88400, Sabah, Malaysia; rbhalan@ums.edu.my; 4 School of Applied Psychology, Social Work and Policy, Universiti Utara Malaysia, Sintok 06010, Kedah, Malaysia; zeyati@uum.edu.my

**Keywords:** personality, traits, criminal, behaviour, systematic review

## Abstract

In addition to social and environmental factors, individual personality traits have intricately linked with maladaptive behaviour. Thus, the purpose of this article was to review the link between individual personality traits and criminality. A systematic review was conducted to obtain information regarding the link between individual personality traits with criminal behaviour in the Sage, Web of Science, APA PsycNet, Wiley Online Library, and PubMed databases. The results indicate that individual personality traits that contribute towards criminality are (i) psychopathy; (ii) low self-control; and (iii) difficult temperament. As an overall impact, the review is expected to provide in-depth understanding of the link between individual personality traits and criminality; hence, greater consideration will be given to the dimension of personality as a notable risk factor of criminal behaviour.

## 1. Introduction

Criminology has become an interdisciplinary field where the focal point of each study has diversely evolved from individual-level to environmental-level risk factors associated with criminal behaviour. As such, individual personality traits constitute one dimension of the bigger picture which has received significant empirical attention in recent decades, especially research linking personality traits to various measures of crime. According to Beaver (2017) [1], personality refers to the stability of individuals in regard to patterns of thinking, feeling, and behaving. In general, personality traits can be categorised into four general combinations, namely (i) high control–high affiliation; (ii) low control–low affiliation; (iii) high control–low affiliation; and (iv) low control–high affiliation [1]. Some empirical research has suggested that high interpersonal control and low interpersonal affiliation are strongly interrelated with antisocial behaviour [1].

The Big Five Model of Personality suggested that five domains largely account for individual differences in personality including (i) extraversion; (ii) openness; (iii) neuroticism; (iv) agreeableness; and (v) conscientiousness [2]. Sleep (2021) [2] stated that low conscientiousness, low agreeableness, and high neuroticism increase aggression, mental distress, and antisocial behaviour among individuals. Similarly, the personality theory constructed by Eysenck (1966) (trait-psychologist) proposes a significant relationship between criminal behaviour and personality variables [3]. Based on the Eysenck personality theory, there are three fundamental factors of personality including psychoticism (P), extraversion (E), and neuroticism (N) [3]. Empirical investigations discover that delinquents score high on the P scale compared to the E and N scales [3]. More specifically, the P scale predicts those involved in violence and sexual crimes, whereas the N scale predicts serious crime and recidivism [3]. Furthermore, a great deal of research has also found that psychoticism is always connected to crime, whereas extraversion is related to younger samples (young offenders/delinquent), and neuroticism is related to older samples (adult offenders) [3]. 

A meta-analysis related to personality and antisocial behaviour has concluded that individuals who commit crime tend to be self-centred, hostile, adhere to unconventional values/beliefs, and have difficulty controlling their impulses [4]. In addition, compared to non-offenders, individuals who commit crimes are less sociable, more aggressive, sensation seekers, and tend to score higher for the neuroticism and psychoticism dimensions [5]. Additionally, Jones et al., (2016) [5], and Cunha et al., (2018) [6], found that individual personality traits represent a predictor of criminal behaviour regardless of gender, race, age, or geographical location. Acknowledging the role of individual personality traits in relation to criminal behaviour, the current study seeks to develop an improved understanding of personality traits to impart significant information to the existing literature in the field of crime studies. 

## 2. Materials and Methods

This review followed the Preferred Reporting Items for Systematic Reviews and Meta-Analyses (PRISMA) guidelines. Keywords such as “personality”; “personality traits”; “individual personality”, “maladaptive behaviour”; “crime”, and “antisocial behaviour” were typed into the Sage, Web of Science, APA PsycNet, Wiley Online Library, and PubMed databases to find the relevant information. 

### 2.1. Inclusion and Exclusion Criteria

Studies that were included in this review are (i) full-text articles; (ii) articles published in Sage, Web of Science, APA PsycNet, Wiley Online Library, and PubMed; (iii) research with at least 20 respondents (to reduce the bias associated with a small sample size; (iv) studies that examine the link between personality traits and criminal behaviour; and (v) articles that were published from January 2016 to June 2021. Conversely, the exclusion criteria in this review were (i) duplicate publication; (ii) articles published before January 2016, (iii) studies with less than 20 respondents (due to small sample size); (iv) non-full-text articles; and (v) articles that do not reflect the link between personality traits and criminality. 

### 2.2. Screening and Selection Process

**Figure 1:**
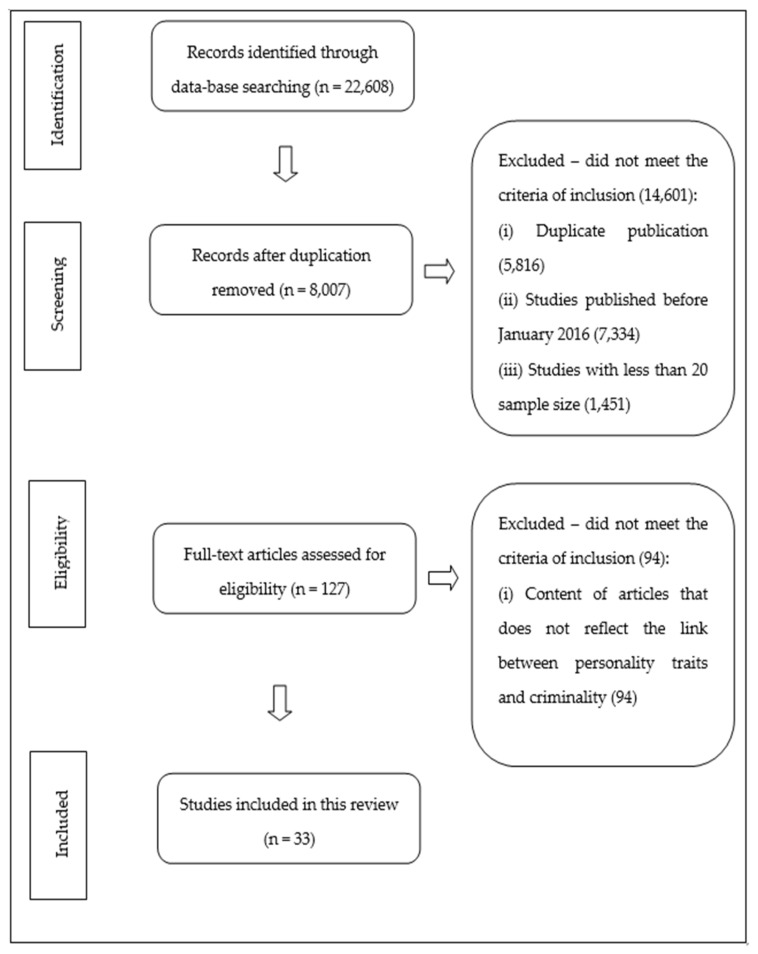
Flowchart of the Preferred Reporting Items for Systematic Review and Meta-Analyses (PRISMA).

For this review, a total of 22,608 sources were found in five well-established databases. A total number of 8007 articles were identified after duplicates were removed. After including other exclusion criteria such as non-full-text articles, year of publication and sample of studies, 127 articles were assessed for eligibility. Furthering this, 94 articles were removed at the eligibility stage since the content of the article did not clearly reflect the link between personality traits and criminality. In the end, 33 full-text articles were reviewed in this study. Figure 1 depicts the flowchart of the systematic review process, whereas Table 1 delineates the summary of articles that were reviewed in this study.

## 3. Results and Discussion

Based on the systematic review, the finding of the study stipulates that there are three major personality traits which contribute towards criminal behaviour, namely (i) psychopathy; (ii) low self-control; and (iii) difficult temperament.

### 3.1. Psychopathy

The term “psychopathy” is commonly used in the global literature on both empirical and theoretical grounds. Psychopathy is a clinical construct associated with emotional and behavioural disturbance, which are considered important risk factors for criminal and antisocial behaviour, criminal recidivism, sexual recidivism, and instrumental violence [7,8,9,10]. Most of the research concerning the measurement of psychopathy has employed Hare’s Psychopathy Checklist (now the Hare’s Psychopathy Checklist—Revised) as the main psychological assessment tool to identify the presence of psychopathic traits in an individual [8]. An individual who scores high for the psychopathy measure (usually > 30 on the PCL-R) is more likely to be short-tempered, irresponsible, egocentric, callous, display superficial charm, frequently violates social norms/values, and be unable to empathise [4,6,7,11]. Similarly, Boccio and Beaver (2016) [11] identified that an individual with psychopathic personality traits have a lower level of self-regulation, are manipulative, impulsive, and unable to feel remorse/guilt. 

Based on the Big Five Model of Personality, scholars have stated that the psychopathy dimension is a mixture of high extraversion, low conscientiousness and agreeableness, and a combination of low and high neuroticism (depression, low anxiety, self-consciousness, vulnerability to stress, high impulsiveness, and hostility). For example, psychopathic criminals tend to commit a wider variety of crimes and are likely to recidivate faster compared to non-psychopathic criminals. In addition, the dominant conceptualization suggests that psychopathy is an inborn condition with a strong genetic component that is further escalated by environmental factors such as adverse childhood experiences (ACEs), traumatic childhood experiences, child maltreatment or parental inadequacy [12,13]. According to Cunha et al., (2018) [6], psychopathy is conventionally conceptualised as a syndrome that remains throughout life and influences different aspects of individual functioning, including their interpersonal, emotional, and behavioural traits. In addition, studies have revealed that psychopathy is more often diagnosed among men (31%) compared to women [4]. Similarly, an incarcerated individual with higher PCL-R scores is more prone to commit violent criminal offenses upon being released from prison [3]. Cunha et al. (2018) [6] also stated that individuals with psychopathic personality traits are unable to form strong emotional bonds with others and struggle to control their temper.

A burgeoning line of research has consistently revealed that the prevalence of psychopathic traits is higher among prisoners compared to general populations [6,7]. Theorist and researchers have more recently contended that approximately 1% of the general population exhibit psychopathic tendency, whereas approximately 15–25% of the prison population display these characteristics [14]. As such, individuals with psychopathic traits begin their criminal activities at a young age and continue to engage in antisocial behaviour throughout their lives [15]. In addition, myriad research outputs from the psychiatry, criminology, neuroscience, and psychology fields of study have shown that psychopathic personality traits are associated with serious juvenile offenders and adult criminals since these individuals are unable to process cues of punishment and rewards [5,6,8,16,17]. Moreover, recent neurocognitive findings unveiled that abnormalities in the amygdala (connected regions of the orbitofrontal cortex) may result in impaired decision making and social functioning, resulting in higher possibilities of engagement in antisocial behaviour [16].

Accumulating evidence stipulates that there are significant differences between types of crime which are commonly committed by a psychopathic female and male [18]. Generally, psychopathic females tend to be less aggressive and rarely repeat their criminal acts compared to males [18]. In addition, in some cases, psychopathic females have a significant level of impulsivity, a trait often associated with borderline personality disorder [18,19]. Furthermore, research related to psychopathic and sexual coercion shows that compared to non-psychopathic individuals, psychopaths are more likely to become sexual offenders (subgroup of rapists) [14]. Similarly, DeLisi et al. (2018) [16] notes that a psychopathic individual also displays severe alcohol and drug use (includes trying a greater variety of drugs and starting to use drugs at earlier age) compared to non-psychopathic populations. 

### 3.2. Low Self-Control

Research examining the underpinnings of crime suggests that low self-control has been consistently linked with involvement in criminal activities [20]. Empirical evidence indicates that low self-control is associated with involvement in delinquency, violence, and antisocial behaviour [21]. According to Boccio et al. (2016) [11] individuals with low self-control are more impulsive, self-centred, prone to risky behaviour, irresponsible, and display volatile temperament. In addition, Brown (2016) [2] stated that individuals with low self-control exhibit six common characteristics. Firstly, those with low self-control tend to be less meticulous, prefer simple tasks that would require little commitment, are short-sighted, and exhibit a lack of self-determination. Secondly, these individuals are easily drawn to the more daring and exciting behaviour/activities. Thirdly, those with lower self-control are impulsive and tend to seek instant gratification, inclined to seize opportunities without considering the dangers/consequences of such behaviours. Fourthly, individuals with low self-control prefer simple activities over concentration-oriented activities such as a long conversation. Fifthly, those with low self-control tend to be less concerned about other individuals’ feelings and have a low tolerance for frustration and conflicts. 

Findings from a broad array of studies have revealed that low self-control is a quintessential predictor of various maladaptive behaviours such as involvement in substance abuse, theft, property offending, and robbery among diverse samples of participants including parolees, jail inmates, and institutionalised delinquents [2,21]. According to Forrest et al., (2019) [21], low self-control increases the probability of an individual engaging in criminal activities when presented with suitable opportunities (mainly because they are unable to ignore or anticipate the potential long-term consequences of their actions). Furthermore, a plethora of studies has agreed that individuals with poor self-control are more likely to engage in a wider range of criminal behaviour such as computer-related crimes, associating with gangs, and participating in antisocial behaviour [20,21,22,23].

Based on the social control theory, Gottfredson and Hirschi argue that females exhibit lower offending frequencies since they are more subjected to stricter enforcement and parental supervision compared to males [21]. The “parented more” variation that exists as a product of parental influence causes females to have a greater ability to self-regulate their behaviour whereas the less effective parenting of male children results in lower levels of self-control, consequently leading to involvement in criminal activities among males [21]. Similarly, Forrest et al. (2019) [21] and Mata et al. (2018) [22] found that gender and type of household (more patriarchal vs. less patriarchal) also influence an individual’s level of self-control. For instance, Mata et al. (2018) [22] note that females growing up in a patriarchal household along with a high level of parental control are less likely to have criminal aspirations. 

A handful of studies have clarified that individuals with low self-control are less concerned with the long-term consequences of their behaviour and are more likely to engage in activities that provide them with immediate gratification, such as shoplifting and fraud-related behaviours [17,20,24,25]. In addition to the negative implications, many studies have indicated that low self-control and a high level of impulsivity is strongly related to socially undesirable behaviour such as smoking and risky drinking [25]. Furthermore, DeLisi et al. (2018) [16] found that low self-control and low moral values escalate intentions to steal and/or fight among individuals who regularly smoke marijuana, occasionally crack cocaine, and drink nearly every day. 

### 3.3. Difficult Temperament

Human development is a complex phenomenon involving the joint influence of socioecological conditions and individual dispositional characteristics. As such, one’s temperament is defined as an individual characteristic which comprises a habitual mode of emotional response to stimulus [17,26]. Foulds et al. (2017) [26] stated that the temperament has been traditionally viewed as an emotional and behavioural characteristic of feelings and presumed to be more biologically rooted by maturation and heredity. Prior research has found that children who throw tantrums will usually react negatively towards people around them, have a low level of bonding with their parents (poor parent–children interaction), and develop various forms of psychopathological problems including antisocial behaviour [29]. According to DeLisi et al., (2018) [16], one’s temperament reflects the baseline differences in the central nervous systems that particularly involve components such as (i) emotionality and mood; (ii) variance in activity level; (iii) withdrawal behaviours; and (iv) self-regulation. In addition, empirical evidence shows that individuals with difficult temperaments experience mood disorders, anxiety disorders, major depression disorders, disruptive behaviour disorders, and drug abuse [17]. Furthermore, Foulds et al. (2017) [26] stated that temperamental deficits also contribute to crime/violence occurrence among adolescents.

Based on the theoretical framework, temperament was divided into nine major dimensions, namely adaptability to the environment; physical activity; approach/withdrawal in response to novelty; regularity of the child’s behaviour (rhythmicity); task persistence; quality of mood in terms of positive/negative feelings; threshold of responsiveness to stimulation; distractibility; and intensity of the reaction [30]. According to Dos Santos et al. (2020) [29], individuals with a low regularity of behaviour (rhythmicity) are more aggressive and delinquent compared to individuals with highly regular behaviour. Furthermore, the result of a study conducted by Nigg (2017) [28] disclosed that girls who scored higher for “adaptability to the environment”; “quality of mood in terms of positive/negative feelings (negative emotional reactivity and low positive affectivity)”; and “approach/withdrawal in response to novelty” (based on the temperament framework) are highly at-risk of engaging in antisocial behaviour. 

Substantial evidence has emerged of adverse childhood experiences (ACEs) (including various forms of neglect and abuse) and temperament factors being significantly associated with conduct problems (relating to poor emotional self-regulation) [17,27,29,32]. The neurobiological model suggests that an early childhood adverse environment and stress regulating systems (autonomic nervous system and hypothalamic–pituitary–adrenal axis) increase susceptibility to severe antisocial behaviour, such as being associated with gang membership, gang delinquency, and gang activities [27,29,31]. Moreover, existing evidence has disclosed that difficult temperament, peer rejection, disciplinary problems, and antisocial peer selection upon school entry also contribute to gang membership among youths [32]. 

Researchers have argued that the home environment, socioeconomic status, and parenting style have a profound impact on child temperament [17,28,29,32]. For instance, Nigg (2017) [28] found that negative parenting practices (inconsistent discipline practice, harsh behaviour, and permissive parenting practice) contribute to behavioural disorders among children. Moreover, some researchers have also begun to acknowledge that parenting roles significantly influence children’s temperament [30,32,33,34,35]. Dos Santos et al. (2020) [31] stated that inconsistent discipline practice by parents and harsh behaviour may accelerate nonaggressive antisocial behaviour (e.g., stealing or frequent truancy) among school-aged adolescents [31]. Furthermore, Dos Santos et al. (2020) [29] also found that a child who constantly receives negative parental feedback for bold behaviour may experience low self-esteem and start to display uncooperative behaviour and incohesive functioning while growing up. In the same line of thought, a great deal of research has revealed that youth with difficult temperaments who grow up in socioeconomically disadvantaged households (marked by poverty, unemployment) and have been exposed to a toxic neighbourhood environment (easy access to criminal gangs, easy access to drugs or firearms) are greatly at-risk of engaging in delinquent behaviour and future criminality across urban and rural contexts [17,33,35].

## 4. Limitations and Direction for Future Research

This systematic review has several limitations. Firstly, information gathered regarding the link between individual personality traits and criminal behaviour was only obtained from the Sage, Web of Science, APA PsycNet, Wiley Online Library, and PubMed databases, and published from January 2016 to June 2021. Thus, there is a possibility that some research published by well-known leading scholars might have been excluded from this review process. Secondly, studies included in this review were limited to articles published in peer-reviewed journals alone without including other resources such as newspapers, letters to editors, or prison reports, thereby limiting the generalizability of the findings. 

Despite the outlined limitations, future research should concentrate on other singular features of individual personality traits such as narcissism, impulsivity, attitude favouring aggression, and Machiavellianism which contribute to criminal behaviour in order to develop diversified treatment protocols based on personality traits. Additionally, future studies should also include mediator factors to allow the in-depth understanding of the process underlying the link between individual personality traits and criminal behaviour. 

## 5. Conclusions

In sum, this review adds to the growing literature in the field of crime-related studies and improves our understanding regarding how personality traits escalate the risk of engaging in criminal activities. Substantial empirical research performed by Gatner et al., (2016) [7] and Nigel et al. (2018) [8] suggested that psychopathy is a robust predictor of criminal behaviour, mainly focusing on instrumental violence. Furthermore, many scholars agree that instrumental violence among psychopathic offenders is significantly determined by the affective traits of psychopathy. Additionally, the inputs obtained through systematic review show that the domain of low self-control predicts a varied range of criminal behaviour. Based on Gottfredson and Hirschi’s social control theory, low self-control contributes to the adoption of deviant values and leads to an individual engaging in various types of antisocial behaviour. Furthermore, a difficult temperament has also been suggested to be one of the key predictors of criminal behaviour, mainly due to the influence of socioecological conditions and individual dispositional characteristics such as sensation seeking, narcissism, Machiavellianism, and sociosexual orientation.

Although the aim of this study was rather academic, the conclusion reached from this finding clearly identifies some significant risk factors for engaging in criminal behaviour. Admittedly, not all individuals with at-risk personality traits are at high risk of becoming delinquents/adult offenders. Therefore, it is essential that the stakeholders and practitioners who work within the criminal justice system to diversify their methods of assessment to identify individuals who fall under the “early onset group”. Furthermore, a proper treatment regimen that matches the result of the rigorous assessment is equally important to promote preventative measures to reduce crime rates in the future.

Through this review, it is transparent that major personality traits such as psychopathy, low self-control, and a difficult temperament can be measured using various scales/inventory or secondary data. Thus, it is suggested that the interventions that aim to reduce the risk of criminality should begin during the early childhood stage since some of the existing evidence agrees that youths usually start engaging in criminal activities after reaching the age of 15 years old [34,35,36]. Moreover, the identification of personality traits regardless of gender is also crucial to initiate appropriate preventative strategies for vulnerable groups such as children, at-risk youths, and adolescents.

## Figures and Tables

**Table 1:** Summary of articles.

No.	Author(s)	Year	Sample	Measures	Findings
1.	Beaver, K.M., Boutwell, B.B., Barnes, J.C., Vaughn, M.G., DeLisi, M. [1]	2017	90,000 adolescents—National Longitudinal Study of Adolescent to Adult Health	Psychopathy, personality trait	Psychopathic personality traits increase the probability of being arrested, incarcerated, and sentenced for both male and female adolescents.
2.	Brown, W. [2]	2016	500 respondents	Low self-control, crime, punishment	Individual with low self-control tend to be less meticulous, prefer simple tasks that would require little commitment, short sighted, and lack of self-determination.
3.	Bo, S., Pedersen, L., Christensen, K.B., Rasmussen, K. [3]	2019	225 male forensic psychiatric patients and prisoners from three treatment institutions in eastern Denmark	Psychopathy, antisocial behaviour	Psychopathic traits increase the risk of violence, especially traits such as impulsivity, irresponsibility, and antisocial behaviour (PCL scales factors 3 and 4).
4.	Traynham, S., Kelley, A.M., Long, C.P., Britt, T.W. [4]	2019	310 incarcerated male U.S. army soldiers and 310 nonincarcerated male army soldiers from Fort Rucker, Alabama area	Psychopathy, suicidal ideation, PTSD, criminal behaviour	PTSD symptoms had a direct effect on incarceration status, and significant indirect effects through suicidal ideation among incarcerated male army soldiers.
5.	Jones, D.N., Hare, R.D. [5]	2016	150 respondents	Psychopathy, lifestyle, antisocial behaviour	Individuals who score high for the psychopathy measure (usually > 30 on the PCL-R) are more likely of being short-tempered and unable to empathise.
6.	Cunha, O., Braga, T., Goncalves, R.A. [6]	2018	52 batterers from Portugal aged between 22 and 70 years old	Psychopathy, criminal behaviour, intimate partner violence	Psychopathy leads to intimate partner violence.
7.	Gatner, D.T., Blanchard, A.J.E., Douglas, K.S., Lilienfeld, S.O. [7]	2016	1742 African American, Caucasian, and Hispanic psychopathic offenders	Psychopathy, criminal behaviour	Psychopathic personality traits show reasonable validity across African American, Caucasian, and Hispanic cultural groups.
8.	Nigel, S.M., Dudeck, M., Otte, S., Knauer, K., Klein, V., Böttcher, T., Maaß, C., Vasic, N., Streb, J. [8]	2018	164 male and female forensic inpatients with substance-related disorders	Psychopathy, empathy, general personality traits, violent crimes of substance-abusing offenders	Substance-abusing violent offenders display a distinct pattern of personality characteristics (associated with high neuroticism, low agreeableness, and low conscientiousness).
9.	Tharshini, N.K., Ibrahim, F. [9]	2020	73 meta-analyses	Psychopathy, low self-control, crime behaviour	Psychopathy construct is associated with emotional and behavioural disturbance, criminal recidivism, sexual recidivism, and instrumental violence.
10.	Tharshini, N.K. [10]	2019	73 meta-analyses	Genetic, personality traits, antisocial behaviour	Genetic and aggression factor strongly leads to antisocial behaviour.
11.	Boccio, C.M., Beaver, K.M. [11]	2016	90,000 adolescents—National Longitudinal Study of Adolescent to Adult Health	Psychopathy, personality trait	Psychopathy is associated with involvement with violent behaviour.
12.	Carabellese, F., Felthous, A.R., Mandarelli, G., Montalbo, D., La Tegola, D., Rossetto, I. Franconi, F., Catanesi, R. [12]	2019	25 Italian female murderers with psychotic personalities	Psychopathy, crime, homicide	Psychopathy is more evident among female homicide offenders who had been abused or traumatized.
13.	Chen, S., Plouffe, R.A. [13]	2020	70 meta-analyses	Psychopathy, crime behaviour	1% of the general population exhibits psychopathic tendency whereas 15–25% of the prisoner population display these characteristics.
14.	Trulson, C.R., Haerle, D.R., Caudill, J.W., DeLisi, M. [14]	2016	100 meta-analyses	Psychopathy, crime behaviour	Individuals with psychopathic traits begin their criminal activities at a young age and continue to engage in antisocial behaviour throughout their lifespan.
15.	Prospero-Luis, J., Moreira, P.S., Paiva, T.O., Teixeira, C.P., Costa, P., Almeida, P.R. [15]	2017	91 male inmates convicted for theft	Psychopathy, crime behaviour	Psychopathic traits are associated with reduced expectancy of negative outcomes and increased expectancy of positive outcomes as a consequence of reoffending among male inmates.
16.	DeLisi, M., Fox, B.H., Fully, M., Vaughn, M.G. [16]	2018	252 juvenile offenders (violence and non-violence delinquency)	Temperament, psychopathy, violence, delinquency	Temperament is the main risk factor for violent and non-violent delinquency.
17.	Edwards, B., Verona, E. [17]	2016	171 community-dwelling women offenders, and 319 women with histories of drug use and/or violence	Sexual risk taking, psychopathic traits, antisocial behaviour	Impulsive antisocial traits associated with sexual risk taking among women offenders.
18.	Verona, E., Vitale, J. [18]	2018	274 meta-analyses	Psychopathy, borderline personality disorder, impulsivity	Psychopathic females have significant level of impulsivity—a trait often being associated with borderline personality disorder.
19.	Ivert, A., Andersson, F., Svensson, R., Pauwels, L.J.R., Levander, M.T. [19]	2018	481 girls and boys aged between 16 and 17 years old	Self-control, antisocial behaviour	Moral values and self-control are significantly correlated with offending among both girls and boys.
20.	Tornquist, M., Miles, E. [20]	2019	253 White, Asian/Asian, American/Asian European, Black/African, American/African European, Hispanic/Latino participants	Self-control, criminal behaviour	Individuals with poor self-control are more likely to engage in a wider range of criminal behaviour such as computer-related crimes and associating with gangs.
21.	Forrest, W., Hay, C., Widdowson, A.O., Rocque, M. [21]	2019	1979 youths between 10 and 30 years old (National Longitudinal Survey of Youth)	Low self-control, risk seeking, impulsivity	High level of risk-seeking and impulsivity contributes towards involvement in criminal activities among youths.
22.	Mata, R., Frey, R., Richter, D., Schupp, J., Hertwig, R. [22]	2018	92 meta-analyses	Low self-control, fraud-related behaviours	Individuals with a low level of self-control engage in activities that provide immediate gratification such as shoplifting and fraud-related behaviours.
23.	Wendel, B.E., Rocque, M., Posick, C. [23]	2020	1744 private college student	Self-control, impulsivity, risky behaviour	Low self-control and high level of impulsivity is strongly related to socially undesirable behaviour such as smoking and risky drinking among college students.
24.	Stifter, C., Dollar, J. [24]	2016	36 meta-analyses	Temperament, antisocial behaviour	Children who throw tantrums will usually react negatively to people around them and have a low level of bonding with their parents; eventually they develop various forms of psychopathology problems, including antisocial behaviour.
25.	Kamaluddin, M.R., Mohammad Shariff, N.S., Mohd Nasir, N.C., Abdul Hamid, A.S, Mat Saat, G.A., Rathakrishnan, B. [25]	2019	140 male adults	Self-control aggression, low socioeconomic status	The result evidenced statistically significant correlation between self-control and aggression levels (r = 0.444, 95% CI: 0.30, 0.57; *p* < 0.001).
26.	Foulds, J., Boden, J., Horwood, J., Mulder, R. [26]	2017	962 general population aged 35 years old and 1025 general population aged 18 years old	Novelty seeking, antisocial behaviour	Alcohol and substance use mediates the association between novelty seeking and antisocial behaviours in early adulthood among general populations between 18–35 years old.
27.	Healey, D.M., Rajendran, K., O’Neill, S., Gopin, C.B., Halperin, J.M. [27]	2016	114 pre-schoolers aged between 3 and 5 years old	Temperament	Higher verbal executive (HVE) is associated with better child functioning when parent-rated effortful control (EC) is high.
28.	Nigg, J.T. [28]	2017	63 meta-analyses	Temperament, aggressive, delinquent behaviour	Individual with low regularity of behaviour (rhythmicity) are more aggressive and delinquent compared to individual with high regularity of behaviour.
29.	Dos Santos, M.A., de Freitas e Castro, J.M., de Freitas Lino Pinto Cardoso, C.S. [29]	2020	69 caregivers, 81 boys	Temperament, morality, parenting behaviour	Low parenting skills and negative moral emotions lead to temperament and morality issues during childhood among boys.
30.	Wolff, K.T., Baglivio, M.T., Klein, H.J., Piquero, A.R., DeLisi, M., Howell, J.C. [30]	2020	104,267 juvenile offenders (mean age of 16, 76% male, 46% Black non-Hispanic, 15.7% Hispanic)	Adverse childhood experiences, gang involvement, temperament	ACEs effect towards gang involvement, substance abuse, and difficult temperament among juvenile offenders.
31.	Perez, M.M., Jennings, W.G., Baglivio, M.T. [31]	2018	64,329 youths	Serious violence, chronic delinquency, adverse childhood experiences	The relationship between childhood adversity and SVC delinquency is mediated by maladaptive personality traits and adolescent problem behaviours.
32.	Tharshini, N.K., Ibrahim, F., Zakaria, E. [32]	2020	306 young offenders undergoing community service order	Demographic profile and perpetrator experience in committing crime	Majority of the young offenders are 20 years old, single in marital status, and employed.
33.	Kamaluddin, M.R., Othman, A., Ismail, K., Mat Saat, G.A. [33]	2017	71 male murderers incarcerated in 11 prisons within peninsular Malaysia	Psychological traits, types of weapons used among the murderers	Aggression and self-serving cognitive distortion are common psychological traits among murderers who use single and multiple weapons to commit crime.
